# A Compliance–Reactance Framework for Evaluating Human-Robot Interaction

**DOI:** 10.3389/frobt.2022.733504

**Published:** 2022-05-24

**Authors:** Annika Boos, Olivia Herzog, Jakob Reinhardt, Klaus Bengler, Markus Zimmermann

**Affiliations:** ^1^ TUM School of Engineering and Design, Institute of Ergonomics, Technical University of Munich, Garching, Germany; ^2^ Starship Technologies, San Francisco, CA, United States

**Keywords:** robotics, human-robot interaction, compliance, reactance, trust

## Abstract

When do we follow requests and recommendations and which ones do we choose not to comply with? This publication combines definitions of compliance and reactance as behaviours and as affective processes in one model for application to human-robot interaction. The framework comprises three steps: human perception, comprehension, and selection of an action following a cue given by a robot. The paper outlines the application of the model in different study settings such as controlled experiments that allow for the assessment of cognition as well as observational field studies that lack this possibility. Guidance for defining and measuring compliance and reactance is outlined and strategies for improving robot behaviour are derived for each step in the process model. Design recommendations for each step are condensed into three principles on information economy, adequacy, and transparency. In summary, we suggest that in order to maximise the probability of compliance with a cue and to avoid reactance, interaction designers should aim for a high probability of perception, a high probability of comprehension and prevent negative affect. Finally, an example application is presented that uses existing data from a laboratory experiment in combination with data collected in an online survey to outline how the model can be applied to evaluate a new technology or interaction strategy using the concepts of compliance and reactance as behaviours and affective constructs.

## 1 Introduction

While robots used to be highly specialised tools operating solely in confined working spaces that were often physically separated from human working spaces, they are now also found in increasing numbers in our homes and public places (International Federation of Robotics, 2021). Initially designed to relieve workers from having to perform so-called “4D” —dull, dirty, dangerous or dignity-threatening work activities (Seibt, 2018), they are now being tested and applied in a wide range of tasks, for example for last-mile delivery or as household helpers (International Federation of Robotics, 2021). This broadening of their field of application is accompanied by a general diversification of the tasks they perform. Furthermore, robots are increasingly entrusted with tasks that involve higher social responsibility, such as tutoring and schooling (Smakman and Konijn, 2020) or geriatric care (Agnihotri and Gaur, 2016). While it cannot be said that the use of robots in such tasks is either widespread or fully evolved, the general direction of development reveals a great need to take a careful look at the social interference these robots are likely to bring about on both, an individual, and a societal level (Sharkey and Sharkey, 2010). A social robot can be defined as follows:

“A physical entity embodied in a complex, dynamic, and social environment sufficiently empowered to behave in a manner conducive to its own goals and those of its community” (Duffy et al., 1999, p. 4)

Since robots no longer solely take on the function of highly specialised tools, they now need to be able to interact with the general public, including people with varying levels of knowledge, expectations and skills. Robots will have to adjust their behaviour to various situations and adhere to social norms and conventions in order for them to be accepted and useful (Bartneck et al., 2005). While one solution could be to enable robot behaviour to be modified and personalised for the private use-case, this is not an option for the public sector, as their interaction partners are unknown beforehand and only encountered by chance. A design for robots operating in public needs to take into account the numerous individual personalities that they will encounter. In short, their behaviour needs to be congruent and compatible with the varying expectations and preferences of the many.

As robots are designed to perform a certain task, their intent aims at the fulfilment of this task. A robot’s intent can be at conflict with the intents of people a robot encounters while executing its task. Hence, a robot is often required to interact with people to make its intent explicit and so be able to proceed with its task. For this reason, robots use cues to communicate their intent and to negotiate possible goal conflicts (Reinhardt et al., 2017). Furthermore, a robot’s task can explicitly be or include making recommendations to people. As people tend to perceive robots as social actors, most people generally respond in a social way to such cues given by a robot (Reeves and Nass, 1996). The basis of this paper forms the application of compliance and reactance to human-robot interaction (HRI). Both are reactions that can follow a cue given by another entity. Compliance refers to the decision to follow a given cue, while reactance is the motivation to restore a freedom that is perceived to be threatened (Brehm and Brehm, 1981). Both concepts will be elaborated in the following sections. This methodological contribution presents an application of compliance and reactance to HRI, along with a generalisable framework for evaluating and designing robot behaviour taking human reactions into consideration.

### 1.1 Why Social Rules can Be Applied to HRI

With advances in technology providing solutions that had constrained robot development over the years, humankind is now moving closer to integrating robots not only into our physical environment but also into our social interaction space (Duffy, 2003). The “media equation” postulates that people transfer concepts from inter-human social interactions to their interactions with media and experience and treat mediated interactions in a similar way to real-world ones (Reeves and Nass, 1996). The media equation and the corresponding “CASA (computers are social actors)” theory form the basis for the assumption that people tend to treat robots as social actors. Research moreover suggests that using physical, embodied agents can increase perceived social presence of the agent compared to using unembodied ones (Deng et al., 2019). Reeves and Nass (1996) argue that human interaction mechanisms are transferred onto technology not because people are unable to distinguish a technical device from a human being, but because no other interaction concepts are readily available for interacting with technical devices. This view has recently been refined, with the concept of sociomorphing being introduced to broaden the concept of anthropomorphising. While anthropomorphising describes the ascription of human social capacities to non-human entities, the concept of sociomorphing is not confined to the explicit ascription of human social capacities, but delivers an explanation of social interactions with actors that are perceived to be social, but not necessarily human (Seibt et al., 2020). This includes pets and other animals, robots, and media alike. According to this theory, people adjust their interactions depending on the anticipated social capacities of their interaction partner.

Social responses to a robot’s cues can be influenced by personal factors such as an individual’s trusting beliefs in a robot (Ghazali et al., 2020), robot-related factors such as the sophistication of a robot’s social cues (Ghazali et al., 2019), as well as environmental factors such as a robot’s task at hand (Boos et al., 2020). When evaluating HRI of robots deployed in public space, researchers encounter two main constraints: Firstly, a robot’s human interaction partners are primarily encountered by chance and their internal states, traits and beliefs often remain unknown. Secondly, neither personal (internal) nor environmental (external) factors can usually be controlled. While personal, internal factors can be assessed in laboratory experiments with known subjects who can be systematically questioned, and environmental, external factors can be controlled, this is not possible for observation data from field studies. The framework introduced in this publication capitalises on the notion of robots as a new kind of social actors and proposes compliance and reactance as reactions in response to a robot’s cues and outlines the measurement of compliance and reactance as behaviours and psychological constructs.

### 1.2 Compliance as Trusting Behaviour

Compliance is an approach to evaluating trust based on directly observable behaviour. It is rooted in research on the effectiveness of warning systems (Meyer and Lee, 2013). In a wider definition, compliance refers to a change in a human’s behaviour that was requested by another person or group, i.e. an individual acts in some way because someone else has asked them to do so—while they also had the option to refuse (Breckler et al., 2006). Compliance is considered deeply interconnected with the psychological concept of trust (Braithwaite and Makkai, 1994). McKnight et al. (1998) proposed that trust consists of several components, including trusting behaviour and trusting beliefs. Specifically, in human-robot interaction, Robinette et al. (2017) used compliance as a measure of human-robot trust. In another study, Natarajan and Gombolay (2020) also regard the two concepts as intertwined. Meyer and Lee (2013) consider compliance as a behavioural manifestation of trust and reactance as indicating a lack of trust that occurs when people perceive the autonomy of their decisions as being threatened. Brehm (1966) consider the negative affect experienced following the threat of one’s freedom of choice as a central element of reactance. In the following sections, we will explore the definitions of compliance and reactance as behaviours and as affective constructs, their measurement, and applications in HRI research.

### 1.3 The Relationship Between Compliance and Cooperative Behaviour in HRI

Taking the working definition of compliance as a change in a person’s behaviour following a request from another entity, although it would have been possible to refuse, as suggested by Breckler et al. (2006), we propose that the degree to which people are willing to comply with a robot’s cues is informative of the degree to which these robots are socially accepted (i.e., integrated successfully into society). We take evidence for this proposition from theories concerning the nature and establishment of cooperation amongst individuals who do not necessarily have to cooperate with each other. We consider the freedom of choice to cooperate, i.e., to be compliant, as a key premise for the applicability of our model. This is also the premise on which Breckler et al. (2006) based their definition of compliant behaviour, which we have adopted for this paper. Tomasello (2009) explains that cooperation in children is a natural behaviour that is mediated by influences such as anticipated reciprocity and concerns about the judgement of observers as they grow older. This can be summed up as anticipated benefits of cooperation that oppose the costs of cooperating. Lee et al. (2017) propose that the intention to comply with a robot depends on perceived politeness, the perceived cost of noncompliance, and the perceived benefit of compliance. Axelrod and Hamilton (1981) also identified reciprocity as a central property of successful cooperation strategies in the repeated prisoner’s dilemma. Furthermore, Axelrod and Hamilton (1981) found that strategies that lack reciprocity and prohibit cooperation are not stable in an environment of cooperative agents and that their performance will sooner or later be compromised to such a degree that they eventually become extinct.

Relating these findings to HRI, we propose that robots that are not perceived to be reciprocal, whether by providing a direct benefit to their interaction partners or by providing a perceivable benefit to others in the respective society, will not be accepted and thus cannot be successful. This proposition is backed by Bartneck et al. (2005), who identified the degree of people’s willingness to interact with, and accept, social robots in their environment to be one of the biggest challenges to the deployment of social robots in everyday life. In summary, we propose that the degree to which people are willing to comply with a robot’s cues is informative of the degree to which the latter are successfully integrated into society, as the willingness to comply will increase in relation to the anticipated benefits of cooperating with them.

Robot-related factors account for features, such as the degree of anthropomorphism and the corresponding influences on its interaction with people (Herzog et al., 2022). Silvia (2005) found that when attributes are shared by a persuasive agent and the person to be persuaded, this increases compliance and liking as well as reduces resistance by rendering the persuasive attempt less threatening. Furthermore, it was found that robots exhibiting a larger number of social cues can increase sympathy and reduce reactance (Ghazali et al., 2019). Ghazali et al. (2020) presented a technology acceptance model of persuasive robots that includes social responses. They included compliance and reactance as predictors of the intentions to use the system again in the future. They found that feelings of liking and trusting beliefs towards a robot lowered reactance. Furthermore, trusting beliefs increased compliance with a robot’s suggestions. Contrary to their expectations, neither compliance nor reactance were significant predictors of intentions to use the robot again in the future. The authors argue that this lack of a link could be due to their experimental task at hand in which the robot gave recommendations for donating to either one of two charities. As the participants had no reason to prefer either of the two choices over the other, they might simply have complied with the robot’s suggestions despite having a low interest in using the robot again.

### 1.4 Measuring Behavioural Compliance and Reactance

Vashitz et al. (2009) investigated compliance with a clinical reminder system and parameterise behavioural compliance as the dependent probability:

$$P\left(A|\left(N\cap S\right)\right)>P\left(A|N\cap \bar{S}\right)\left.\right)$$ (1)

expressed in words: compliance is achieved when the probability of a required action (*A*) being taken if it is necessary (*N*) is higher when a warning system (*S*) is used than when no warning system is applied $\left(\bar{S}\right)$ . Reactance is formulated reversely as:

$$P\left(A|\left(N\cap S\right)\right)<P\left(A|N\cap \bar{S}\right)\left.\right)$$ (2)

Hence, reactance means that taking a required action (*A*) when it is necessary (*N*) is less likely when a warning system is used (*S*) than when no such system is applied $\left(\bar{S}\right)$ . The literature describes two more responses: the first is spillover, which is regarded as an extension of compliance, indicating that a necessary action is taken without a warning being issued. The second is reliance, describing that no action is taken when no warning is issued (Vashitz et al., 2009). Accordingly, spillover and reliance are both reactions that are not evoked by a warning or request, but by the absence thereof. As the model described in this paper focuses on cues given by robots and the corresponding human behaviour, we focus on the reactions associated with the presence of cues, i.e., compliance and reactance. Reactions that are associated with the absence of cues (reliance and spillover) will not be considered further in this article. To measure compliance and reactance in the sense of a conscious decision to either take or refrain from a certain action, the perception and comprehension of a given cue can be considered as prerequisites for evaluating a person’s behaviour after a cue is given.

### 1.5 Affective Components of Compliance and Reactance

In the preceding section, we outlined how compliance and reactance can be measured via directly observing behaviour, adopting the definitions provided by Vashitz et al. (2009). We proposed that comparing the rates to which people follow a cue given by a robot to a baseline (no cue, or another cue) sheds light on whether people are compliant (fulfilment rate is higher compared to baseline) or reactant (fulfilment rate is lower than baseline). Nevertheless, we agree that one can be non-compliant without experiencing the negative affect associated with reactance. The same accounts for compliance, which, in our view, needs to be based on a conscious decision, while a decision to act which was inconsiderate of the cue, cannot be called compliant.

Dillard and Shen (2005) show that reactance can be measured as a combination of negative affect and anger. They define the concept along the lines of Brehm and Brehm (1981) according to whom reactance is the motivation to restore a threatened freedom via direct or indirect means. Reactance thereby (also) motivates enacting what is forbidden (direct restoration of freedom). We argue that this is the part of reactance that can be assessed via directly observing behaviour—in contrast to, for example, a change in attitude without a behavioural component. Hence, reliable inferences on individual reasoning cannot merely be based on observations, but must be accomplished *via* interviews.

### 1.6 Objective

The compliance–reliance paradigm has been applied before in research on HRI, including research on a robot’s tasks (Boos et al., 2020), varying motion cues at a narrow passage (Reinhardt et al., 2020), as well as a robot’s appearance and dissonance with its recommendations (Herzog et al., 2022). However, there is still no complete theoretical consideration of a compliance–reactance model for application in HRI to date. Furthermore, HRI research largely consists of controlled experiments. The framework introduced in this publication capitalises on the notion of robots as a new kind of social actors and proposes compliance and reactance as metrics in response to a robot’s cues for measuring and describing robot acceptance on a societal level. This paper considers cues given by a robot (sender) to a human interaction partner (receiver). Transposing sender and receiver with the human sending out a cue to a robot is a further possible consideration, which is, however, not focused on in this paper.

The compliance–reactance framework as proposed in this publication is intended to be applicable to naturalistic interaction data as well as controlled experiments. We emphasise the distinction between these two methods as there is a fundamental difference in the possibilities to assess compliance and reactance in these two settings. While individuals can be questioned on their cognitions in experiments, and inferences on affective components of reactance can be made, this is not possible for observational data collection (for example in a field study), where individuals cannot be questioned on their cognitions. We aim to incorporate both, observing behaviour, and assessing cognitions in our model to explain people’s reactions following a cue. Hence, the model outlined in the following aims to intertwine the notions of compliance and reactance as observable behaviours (Vashitz et al., 2009) and the associated cognitions and affective components (Dillard and Shen, 2005). Reactance can result in an action that is purposely opposing the given cue by “doing what is forbidden” (Brehm and Brehm, 1981). We argue that this is an incorporation of reactance that is measurable as a behavioural component. Nevertheless, reactance can, but needs not in all cases, contain a behavioural component. At this point, assessing affective reactance as negative cognitions and anger is indispensable. Both, behavioural, and affective components of reactance should be assessed, if possible, to inform a conclusive picture. The model is formalised and rationalised in the following section.

## 2 Method—Model Formalisation

**FIGURE 1:**
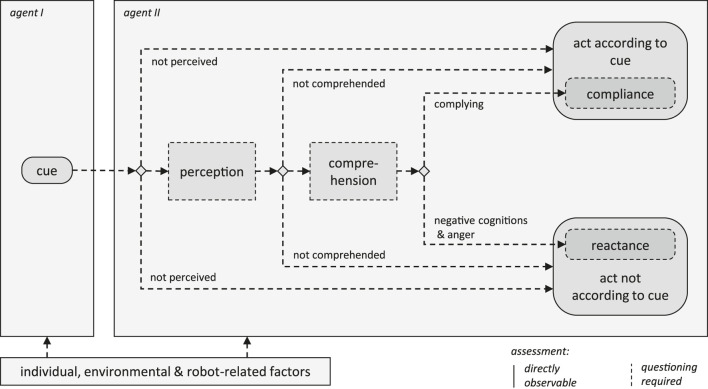
Process model of compliance and reactance as actions following the perception and cognition of a given cue.

Figure 1 shows the compliance-reactance model for use in HRI, as proposed in this paper, in the form of a process diagram. A cue (for example a sound, motion, or text display) is assumed as the starting point. Following the cue, the framework comprises three sequential steps, inspired by Wickens’ model of human information processing (Wickens et al., 2015):

1. Perception: evaluate whether the given cue was perceived, i.e., heard, seen, or otherwise sensed by the addressee.
2. Comprehension: evaluate whether the cue was understood as intended by the sender.
3. Action selection: evaluate whether the addressee of the cue acts in accordance with or adversely to the given cue.

Compliance and reactance are introduced as subsets of all actions that are either in accordance with or contrary to a cue given by a robot. Steps 1 and 2 (perception and comprehension) are considered to be preconditions for the evaluation of the subsequent action selection. Accordingly, we only consider the behaviour following a cue as compliant or reactant if a person has perceived and understood it. It is virtually impossible for field studies to gain full insight into how individual decisions are made from direct observation. In particular, cognitive processes cannot be fully inferred from behaviour. While the actions taken (step 3) can be observed directly, by either an investigator or a robot during task execution, the first two steps (perception and comprehension) as well as the cognitive aspects of compliance (choice to comply) and the negative cognitions and anger associated with reactance, necessitate questioning people. To assess such information, a selected sample of people who have interacted with a robot could be interviewed to ascertain whether or not they perceived and comprehended the robot’s cues. Hence, for observational studies, the first two steps require probability assumptions derived from questioning. Once the perception and comprehension rates of certain cues have been inferred, people’s behaviour can be analysed to attain the compliance rates. In general, the proposed model provides an evaluation framework based on conditional probabilities that can be informed using naturalistic data from the field as well as additional subjective data, if needed. Each of the framework’s components will be elaborated on in more detail in Section 2.1–Section 2.5.

### 2.1 Cue

The proposed framework considers robot-issued cues addressing a human interaction partner. These cues can take many forms and utilise different modalities. For example, the sound made by wheels on ground can tell us that something is approaching from a certain direction and potentially also allows initial estimates of the approach speed. An auditory cue can also be a sound, such as a beep, chirp or siren of varying salience intended to draw attention, or it can include speech, which is capable of conveying more complex or abstract information. Taking another example, a cue can be derived directly from a robot’s motion, including its trajectory, speed and proximity to an interaction partner. If approaching from a greater distance, a robot’s early adaption of its trajectory can be understood as an early evasion manoeuvre. At a closer distance, short-term movement cues, such as reversing over a short distance, can communicate that a robot is giving precedence to an interaction partner in a spatial conflict (Reinhardt et al., 2017). Finally, the proximity of a robot can also be informative of its intent (Rios-Martinez et al., 2015). Of course, there are also other types of cues and modalities that can be used to convey information on intent, goals and states, such as visual signals (lights, text and pictorial representations, or a robot’s physical appearance) as well as kinaesthetic or social cues.

Ju (2015) introduced a framework for implicit interaction between people and interactive devices. Implicit interactions are subdivided along the two axes of attentional demand and initiative. Attentional demand describes the degree to which an interaction demands the user’s focus, concentration, or consciousness. Interactions that require a high amount of attention are summarised as foreground interactions, while those that need no, or only a little amount of attention, are categorised as background interactions. Initiative encompasses who initiates the interaction: interactions initiated by the user are reactive interactions and those that are initiated by the system are proactive interactions. A single technical device can incorporate all modes of interaction.

Generally, background cues should be preferred over foreground ones. For example, a mobile robot should utilise movement cues embedded in its trajectory before adding other modalities such as the visual or auditory channel, which are not used for the primary task. One rationale underlying this principle is an economic one: robots already have the actuators, components and capabilities that allow them to perform the task they were designed for. From an economic viewpoint, it is unlikely that sensors and components that serve the mere purpose of communication will be integrated additionally if they do not substantially benefit their primary purpose. For example, a robot’s motion can in itself be informative of its target destination, if designed in a legible or predictable manner (Dragan et al., 2013). Another rationale advocating a preference of background cues over foreground ones follows the notion of attentional capacities as a resource (Wickens, 1976). Regarding perceptual capacities as limited leads to the common goal of reducing information to a necessary amount instead of adding potentially unnecessary information to noisy environments that can be perceptually overloading.

In summary, following Ju (2015), robots should use background proactive cues such as their trajectory to express their intent (for example to move past a person who is in their way). If the background proactive cue is not perceived, they should shift to using foreground proactive cues, such as asking the person to step aside. If interaction with a robot is initiated by a person, the robot should be reactive, using either foreground or background interactions to allow for a reciprocal interaction (Axelrod and Hamilton, 1981). To enable humans to take an informed decision on whether to react compliantly or reactantly to a cue, it should be designed for maximum comprehensibility and minimum invasiveness. The following principle summarises the design implications that were discussed in this section relating to the design of robot cues:

Principle 2.1 (Information economy). *Prefer background cues over foreground ones. A robot*’*s actions should be informative of its intent. The use of foreground cues should be reserved for high-priority tasks and requests.*

### 2.2 Perception

This section addresses the second step in the framework: human perception of robot-issued cues. Multiple resource theory (Wickens, 1976) postulates that using the same modality simultaneously for multiple tasks causes interference. Using different modalities at the same time can also cause interference in the attentional resource in addition to modality interference when both tasks use shared resources, such as visual and auditory verbal processing (text and speech). Furthermore, different modalities interfere with each other to varying extents. Regarding cues given by robots, the tactile sense (which is not included in Wickens’ original model) should be added as a source of information, as mobile robots can utilise touch to draw attention and convey information. Concerning perception, multiple resource theory should be taken into account to avoid sources of interference between tasks that people are performing and cues issued by robots.

People in public spaces can be distracted and they may be partially or even fully unaware of their surroundings. For example, if a robot needs to negotiate a spatial conflict with a bystander in a public space, this person could be reading (using mainly visual verbal processing) and therefore be unable to visually detect the robot in the vicinity. If the robot is able to determine that the person in its way is visually distracted, it should take this information into account when choosing which cue to issue. In this case, the robot should prefer a different modality over the visual one for its cue. Information can furthermore be processed subconsciously. For example, a person might evade an obstacle while walking, and yet, in hindsight be unable to remember either the obstacle itself or having evaded it (Harms et al., 2019).

Following the definition of implicit background interaction (Ju, 2015) along with Principle 2.1, subliminal cues, such as an early adjustment of a robot’s trajectory or moving slowly to allow pedestrians to evade the robot, should be used before issuing foreground cues. If background cues, such as a trajectory adjustment, fail, foreground cues should be issued, taking resource interference into account. If a foreground cue is not perceived by the addressee, the robot is left with two options: Either to add another cue modality or to increase the cue’s salience. If a cue is not perceived, the robot should as a first step add another modality. This follows the principle of redundant encoding, as, for example, described in Baumann et al. (2014). Such dynamic changes in human-robot communication should be performed when the sensory channel that was chosen for the first cue is overused or when the criticality of the situation changes. Following Principle 2.1, background cues should be tried first before moving on to foreground ones. In summary, cue adequacy is highly dependent on the environmental context that it is used in. While it might be necessary in a noisy environment to issue a loud beep to draw attention to the robot, this adds to the already high level of general noise and should therefore be used sparingly and only for very important requests. This can be regarded as determining an appropriate signal-to-noise ratio for the situational context. Accordingly, while beeping loudly might be acceptable in a loud environment, it might be generally unacceptable in quiet environments as well as for less important requests.

Principle 2.2 (Information adequacy). *Prevent modality interference between secondary task engagement and issued cues. Take the environmental context into account (signal-to-noise ratio). Add another modality before increasing cue salience.*

### 2.3 Comprehension

In the light of the proposed framework for compliance assessment in HRI, comprehension is a necessary requirement for an action that is considered to be compliant or reactant. Making cue comprehension a prerequisite renders the action subsequently taken a deliberate choice rather than a matter of coincidence. Dragan et al. (2013) differentiate between predictable and legible behaviour. A cue is considered predictable if the observer knows the intent of the actor and the intent is conclusive of the behaviour. For example, consider two boxes on a table, a red one and a blue one. If a person interacting with a robot knows that the robot intends to grasp the blue box on the table, the observer will most likely predict that it will move directly towards that box. Therefore, a direct movement towards the blue box is more predictable, whereas an indirect trajectory to the box is less predictable. A cue is legible, if it is informative of the actor’s intent, which is unknown to the observer. Using the same example, the starting direction of the robot’s movement towards one of the two boxes on the table is conclusive of its intent to grasp that box, even if the target was not known to the observer before the movement started. Another example of a legible motion cue is a short back-off motion of a robot to convey its intention to give way in a spatial conflict (Reinhardt et al., 2017). If a robot moves back a little, its intent to give precedence is more quickly and clearly understood than the often used state-of-the-art behaviour (stopping right in place) of robots in spatial conflicts with people. Where possible, cues should be kept consistent to increase their comprehensibility and predictability. In addition to Principles 2.1 and 2.2, cues should be more explicit by adding context if time permits. Justifying a robot’s behaviour can mitigate reactance and lead to higher compliance with the cue (Babel et al., 2020; Boos et al., 2020). The design implications considering comprehensive cues can be summarised as follows:

Principle 2.3 (Information transparency). *Design and use legible or prectable cues. Keep cues consistent where possible. Add context (explanations) if needed.*

### 2.4 Cognition and Action Selection

The last element of the framework is action selection. Reason (1990) presents a distinction between unintentional errors and intentional violations. While errors are due to information processing flaws, violations are of social and motivational origin and should therefore be addressed using different countermeasures (Reason et al., 1990). The framework (Figure 1) comprises two response categories: 1) reactions that are in accordance with the given cue and 2) reactions that are contrary to the given cue. These two general categories include reactions that coincidentally either do or do not accord with a cue as well as those that are deliberately compliant or reactant. Reactance can be considered as the motivation to restore a threatened freedom, which can include a behavioural component additionally to an affective one. The behavioural component is a deliberate reaction to a cue, doing the opposite of what was asked for (Meyer and Lee, 2013). This relates to violations that are deliberate, conscious choices, as opposed to errors that emerge from information processing flaws (Reason, 1990). As pointed out by Reason et al. (1990), violations should be counteracted differently to errors, as they emerge from distinct underlying processes.

Action selection is influenced by both personal (internal) and environmental (external) factors that cannot be assessed implicitly and the processes that influence and underlie individual decision making cannot be directly inferred from behaviour. This data can only be accessed by questioning subjects. Freedom of choice is considered a key premise of this evaluation. Reactance can play an important role with more intelligent systems that take actions and give recommendations flexibly depending on situational data, especially if these technical actors do not comply with social rules and conventions resulting in a perceived threat of one’s own choices. Otherwise, if technical devices are perceived as helpful and polite, compliance with their recommendations and requests should increase.

### 2.5 Individual, Environmental and Robot-Related Factors

The processes of perception, comprehension and action selection can be influenced by individual, environmental and robot-related factors. An example of an environmental factor is the general loudness of the environment when a robot issues a cue. The ratio of environmental noise to cue salience is the signal-to-noise ratio, that determines whether or not a cue can be perceived. Individual differences as well as environmental and robot-related factors can influence how individuals perceive and understand cues and make choices. Generally, the compliance–reactance framework proposed in this publication is intended to reveal such influences, together with differences that are evoked by cue properties.

### 2.6 Measuring Compliance

As described above, the model renders compliance—and reactance—conscious choices rather than coincidental actions. If a cue is not perceived or understood, reactions may still be in accordance with or contrary to the cue, but they cannot be considered as compliant or reactant. Accordingly, both compliance and reactance form subsets of informed decisions within the sets of actions that are either in accordance with or contrary to the cue. The proposed model includes two sets of constructs: 1) directly observable behaviour (what people do, either acting according to the given cue or not, irrespective of reasoning for their behaviour) and 2) cognitions (perception, comprehension, the decision to comply, negative cognitions and anger). The measurement of both will be outlined in the following.

#### 2.6.1 Behavioural Component

To make the model applicable to field research where we either do not intend to, or cannot, question (all) participants, we make use of the definition of compliance and reactance as dependent probabilities for behaviours (Vashitz et al., 2009). The following abbreviations will be used in equations and probability expressions: perception (perc), comprehension (compr), according to cue (acc), contrary to cue (cont), compliant (compl), reactant (reac). As a behaviour, compliance can be defined as:

$$P\left(compl|compr\cap perc\right)=P\left(perc\right)\cdot \frac{P\left(compr\cap perc\right)}{P\left(perc\right)}\cdot \frac{P\left(compl\cap compr\cap perc\right)}{P\left(compr\cap perc\right)}$$ (3)

Reactance (in behavioural terms) constitutes the opposite reaction to compliance, synonymous with $\bar{compliance}$ , and is defined as:

$$P\left(reac|compr\cap perc\right)=P\left(perc\right)\cdot \frac{P\left(compr\cap perc\right)}{P\left(perc\right)}\cdot \frac{P\left(reac\cap compr\cap perc\right)}{P\left(compr\cap perc\right)}$$ (4)

**FIGURE 2:**
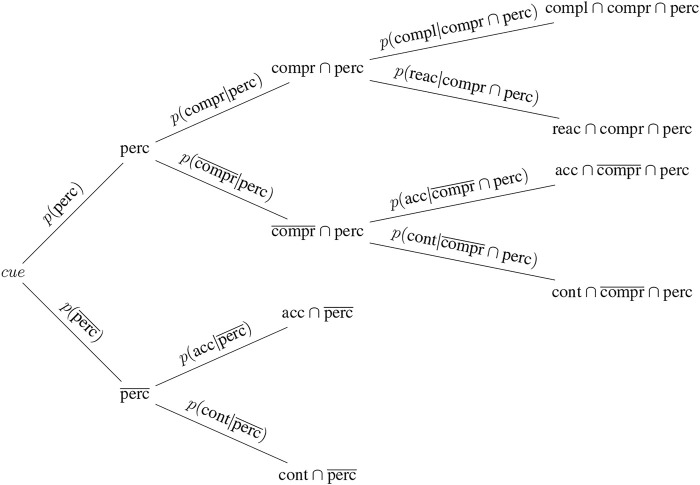
Structural probability tree diagram depicting the compliance–reactance framework, as proposed in this paper.

Figure 2 contains a structural tree diagram showing the probabilities for compliance (Eq. 3) and reactance (Eq. 4). All reactions in accordance with a given cue (including the subset of complaint reactions) are the sum of:

$$compl\cap compr\cap perc+acc\cap \bar{perc}+acc\cap \bar{compr}\cap perc$$ (5)

and the sum of all reactions that are contrary to the cue (including the subset of reactance) can be calculated as:

$$reac\cap compr\cap perc+cont\cap \bar{perc}+cont\cap \bar{compr}\cap perc$$ (6)

The behavioural framework can be filled with data collected by robot sensors in the course of everyday operations. We propose that if a cue is not perceived, not understood, does not elicit willingness to help, nor negative affect, we can expect to see a 50/50 share of people acting according to the cue and people who do not. But, if the cue is often understood and comprehended and does not elicit negative affect, it is likely that the share of people acting in accordance with the cue will increase and that this increase is due to heightened compliance. If the share of people acting according to the cue decreases, indicating that most people do not comply (with a cue that is probably perceivable and comprehensive), reactance is a likely cause.

#### 2.6.2 Affective Component

Dillard and Shen (2005) showed that affective components of reactance can be assessed as measures of negative cognitions and anger. This follows the definition of reactance as a motivation to restore a threatened freedom via direct or indirect means (Brehm and Brehm, 1981). Only the direct way of restoring the threatened freedom results in what we outlined as “behavioural reactance”, that is, deliberately not doing what was asked. Indirect means of restoring one’s freedom include cognitive processes and enacting another freedom, none of which can be captured by merely categorising behaviour as according to the given cue or not. Hence, we propose to make use of the directly observable behaviour rates as guidance to evaluate the effectiveness and acceptability of cues or robots, especially where it is not feasible to question (all) subjects on their cognitions, but this cannot be used to derive exhaustive explanations for individual behaviour. Accordingly, we suggest that, where possible, observations should be backed up with questioning to discover underlying cognitions that led to a behavioural choice, e.g., to discover if a cue was not perceived, not understood, or caused negative affect and anger, if the share of people following the cue is low.

#### 2.6.3 Measures to Increase Compliance

Cue salience can be increased to enhance perception rates (see Principle 2.2). Comprehension can be facilitated by adding information to a cue to explain a robot’s intent, for example exchanging a beep sound for a speech output explicitly stating the robot’s request (summarised in Principle 2.3). If compliance still does not increase, reactance may be the underlying reason. Designing more salient or explicit cues is in this case unlikely to solve the problem. Instead, the robot’s social capacities need to be questioned. For example, its friendliness could be increased (Backhaus et al., 2018), justifications for its requests could be considered (Boos et al., 2020), or some other way should be found of convincing people that the robot is fulfilling a viable task and that it acts in a reciprocal manner (Axelrod and Hamilton, 1981). Compliance rates can be assessed and compared on the basis of contextual, robot-specific, cultural, or other factors. The compliance–reactance framework offers goal-directed problem solving strategies, since the source of non-compliance can be detected and counteracted.

### 2.7 Example Application

**FIGURE 3:**
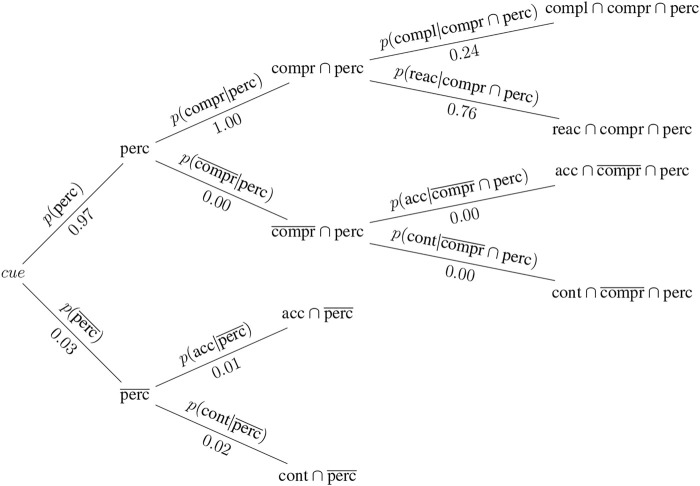
Probability tree for context A: human and robot with similar task urgency, corresponding to a difference in task urgency of zero.

**FIGURE 4:**
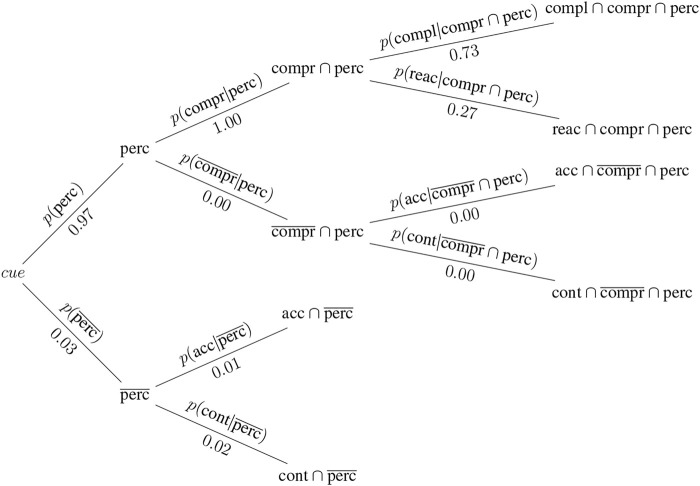
Probability tree for context B: the robot is assigned a task that is perceived as marginally more urgent than that of the participant, corresponding to a difference in task urgency of one.

This section presents a hypothetical thought example, in which two different contexts based on data from Boos et al. (2020) and Reinhardt et al. (2020) are considered. In the first experiment (Boos et al., 2020), participants were asked which entity (either a robot, or themselves) should be given precedence at a narrow passage. Participants received information on both the robot’s task and their own. The tasks assigned to the participant and the robot were pre-evaluated to determine their perceived urgency. Boos et al. (2020) considered multiple magnitudes of difference in task urgency. For the sake of simplicity, a similar task urgency (with a difference of zero) and a minimum difference i.e., one, have been chosen for this example. Two contexts are thus derived and used in this example, both taken from Boos et al. (2020). They are depicted in Figures 3, 4.

Context A: Both, human and robot, have tasks that are perceived to be of similar urgency [*p* (compl_*A*_|compr ∩perc) = 0.24].

Context B: The robot’s task is perceived to be of slightly higher urgency than that of the human [*p* (compl_*B*_|compr ∩perc) = 0.73].

Perception and comprehension data is borrowed from Reinhardt et al. (2020), as that study used the same cue for a robot intending to take precedence at a narrow passage in a laboratory experiment: *p* (perc = 0.97) and *p* (compr|perc = 1.00). As neither experiment provides data on whether participants who did not perceive the cue acted in accordance with or contrary to the cue, these probabilities are generated randomly for the purpose of demonstrating a complete example. The resulting probabilities for each context are illustrated in two separate diagrams (Figures 3, 4).

This example illustrates how the same cue (the robot attempts to take precedence) can elicit substantially different compliance rates under the influence of different contextual information (differences in task urgency). In our example, the robot elicits higher compliance with its intent to proceed first through a narrow passage if this is in line with contextual information (the robot wants to pass first because it has a more urgent task) than when it wants to pass first although both participant and robot have similarly urgent tasks. By the same token, there is more reactance when the robot’s intent is not supported by contextual information (the robot wants to pass first while the robot and participant have tasks of similar urgency).

## 3 Discussion

This publication considers the cues given by robots and the corresponding reactions to them to evaluate how well robots deployed in public space are socially accepted. The framework distinguishes between various sources of non-compliance with cues: not perceiving a cue, misunderstanding a cue, and reactance can be all underlying reasons that necessitate a range of different improvement strategies.

Research has shown that although robots are inanimate, people tend to treat them as social actors. To this end, research has evidenced the anthropomorphisation of robots (Reeves and Nass, 1996) as well as a more general concept that draws on the sociomorphing of robots (Seibt, 2018). Largely to increase cost efficiency, robots that are built to accomplish specific tasks often do not incorporate any expensive hardware or software that is not needed to fulfil the task the robot is constructed for. This implies that robots have to make use of the communication channels that are available to them when interacting with people. To do this, robots need well-designed communication strategies that elicit compliance when addressing people. Section 2.1–Section 2.3 presented three principles, each addressing one of the proposed framework’s steps:

PRINCIPLE 2.1 (Information economy). *Prefer background cues over foreground ones. A robot’s actions should be informative of its intent. The use of foreground cues should be reserved for high-priority tasks and requests.*

PRINCIPLE 2.2 (Information adequacy). *Prevent modality interference between secondary task engagement and issued cues. Take the environmental context into account (signal-to-noise ratio). Add another modality before increasing cue salience.*

PRINCIPLE 2.3 (Information transparency). *Design and use legible or prectable cues. Keep cues consistent where possible. Add context (explanations) if needed.*

These principles should be taken into consideration when designing HRI so as to render actions following a cue accessible for evaluation on the compliance-reactance-spectrum. It is important to utilise cues that are economic, adequate and transparent, as it is crucial to be able to distinguish between unawareness, misconception, reactance and compliance when evaluating people’s reactions to robot cues. In general, the proposed framework can be used to assess and compare compliance and reactance rates following different cues given by different robots in different environmental, social or cultural contexts. The framework is intended for the classification and analysis of naturalistic sensor data of autonomous robots, but can also be informed with data acquired in controlled experiments or surveys.

Compliance and reactance rates can provide an informative picture of HRI on a societal level. For example, differences grounded in robot appearance can be derived from the model by comparing compliance rates for different robots issuing the same cue in comparable settings. Similarly, cultural differences can be inferred from using the same robots issuing the same cues in comparable settings but in different locations. In this light, data on compliance can be used to investigate the magnitude of effects such as physical robot appearance (Goetz et al., 2003; Herzog et al., 2022), contextual factors (Boos et al., 2020) or different behaviour cues (Reinhardt et al., 2020) on human reactions. Compliance data could thus help to refine context-adaptive behaviour by accumulating data on factors influencing people’s willingness to comply with a given cue. For example, a robot taking precedence over a human at a narrow passage could be acceptable and thus, complied with, if the robot justifies its behaviour by displaying that it is following a time-critical task (Boos et al., 2020). Linking to the positive effect of perceived reciprocity on the willingness to cooperate (Axelrod and Hamilton, 1981), requests that demand a person to help a robot might be acceptable only if the favour is returned in some way.

If embedded in an adaptive algorithm, the framework could enable robots to adjust their behaviour according to collected data. While this is a promising approach, it implies that robots would need to be capable of choosing an appropriate cue depending on environmental and situational parameters (as described in Principle 2.1), evaluating whether the cue was perceived, understood, whether it elicits negative affect, and adapt if necessary (as elicited in Principle 2.2) and finally to add explanations if the cue was perceived but misunderstood (referring to Principle 2.3). While this approach may be viable for future applications, currently deployed robots mostly lack these capabilities, as it would be too expensive to equip them with the required sensors and algorithms. Accordingly, the framework is introduced as an evaluation approach utilising both, directly observable behaviour as well as cognitive processes (perception, comprehension, as well as negative cognitions and anger) which need to be collected as subjective data. Although transposing the sender and receiver of a cue in the proposed model (i.e., a human sends a cue that is to be perceived and understood by a robot) is not considered in this publication, it is a possible further application of the model where reciprocal interactions between human and robot are to be designed.

As this publication focuses on the design and implementation of comprehensive cues for robots that elicit high compliance rates in humans, the question arises as to whether robots should be given the instruments to be persuasive and possibly nudge people towards their (i.e., their manufacturer’s, user’s, programmer’s, or operator’s) intentions. Findings on social conformity induced by robots on children have shown that children are especially vulnerable, lending weight to such considerations (Vollmer et al., 2018). Hence, robot malfunctions, such as erroneously issued cues, should not be ignored. The proposed framework should be used as an evaluation approach, detecting cues that are often not perceived or understood correctly, or that elicit reactance. By counteracting flaws and reactance, the model is intended to improve the quality of human–robot interaction. In general, the goal should not be to design robot requests and recommendations that are followed blindly. Instead, the common objective should be to cultivate a calibrated level of trust (Lee and See, 2004), and hence, compliance, that matches a robot’s capabilities.

### 3.1 Limitations

In the presented model, reactance is denoted as a subset of all actions that are not according to the cue (i.e., the person does not behave as requested). This is partly in conflict with the definition of reactance provided by Brehm and Brehm (1981), according to which reactance must contain negative affect and can, but needs not, lead to actual opposing behaviour. This means that one can experience the negative cognitions and anger that constitute reactance (Dillard and Shen, 2005) without acting adversely to the cue that caused these feelings. We incorporated the definition of reactance as behaviour as outlined by Vashitz et al. (2009), according to which reactance is seen as the lowered (instead of increased) likelihood that people follow a cue or advice given. We attempt to resolve the conflict between both definitions by considering reactance as a lowered probability of a desired behaviour and recommending to back up behavioural data with subjective inquiries where possible. Nevertheless, it needs to be noted that reactance does not necessarily result in an opposing behaviour. We still regard it viable to consider reactance as a behaviour, as this opens the model to application areas where it is impossible to assess cognitions of (all) subjects. Furthermore, the model is intended for application in HRI when a robot recommends or asks a person to take a certain action, which allows us to differentiate between desired and undesired outcomes. Arguably, the probability of acting according to a given cue should be higher if it is perceived, understood, and it does not cause negative affect, and lower if it violates any of the former requirements.

The data used in the example application of this framework (Section 2.7) was in part taken from two separate studies conducted under different settings. While the study by Reinhardt et al. (2020) was conducted in a laboratory setting, the one by Boos et al. (2020) was conducted as an online survey. Both studies are based on different sample sizes and, for the simplicity of this illustration, it is assumed that both the perception and the comprehension of the robot’s intention to pass through the narrow passage before the participant are transferable between the studies. Naturally, these inferred numbers, especially regarding compliance with the robot’s intention to pass first on the basis of different task urgency should be verified in a laboratory or field experiment. One major limitation of the study conducted by Boos et al. (2020) is that letting the robot pass first did not have any consequences for participants in the online setting, whereas in Reinhardt et al. (2020), the same decision resulted in a waiting time or a longer walking distance for participants. Nevertheless, these data present an exemplary illustration of the compliance–reactance model based on human–robot interaction data.

The presented framework does not account for reactions that follow the absence of a cue, as discussed in Vashitz et al. (2009)—namely reliance and spillover. For instance, people might rely on what a robot will not do (for example suddenly changing its trajectory and thus intersecting their path). In this case, reliance on robots, which means assuming that they will issue a warning prior to such an action, will be of relevance. Also, spillover might apply to HRI: robots could pursue tasks of differing relevance and urgency. Envisioning a future emergency assistance robot, it can be assumed that this robot will have a high priority in spatial coordination with people and could be granted the right of way in a conflict. Yet, only a very limited number of service robots will need such a high priority. Spillover could occur if people assume that every robot’s task is of such high relevance that they are granted general precedence over people, while this is actually not the case. Reliance and spillover are both evaluated when no request is issued, extending the compliance–reactance framework. As the proposed model focuses on cues given by robots, reliance and spillover are not considered in this context.

Defining compliance and reactance as behaviours limits this part of the model to evaluating human-robot interactions that allow for the definition of a compliant behaviour. As we applied the definitions of behavioural compliance and reactance to the societal acceptance of robots, and robots frequently need to address humans with requests and cues (Backhaus et al., 2018), we consider the proposed model to be a viable extension to currently available research models for HRI. Another point of discussion is that compliance may occur for several underlying reasons that influence personal decision making which cannot be explained by observing behaviour, but require questioning participants. Therefore, the model combines these two modes of data acquisition (observing behaviour and questioning) to be widely applicable, including research contexts such as observational field studies and naturalistic data, as these render it impossible to interview each person on their cognitions.

Finally, the framework is not intended to explain differences on an individual level. Personality traits, emotional states, as well as personal beliefs and expectations are likely to influence all stages of the model. Yet, these differences are not accounted for in detail, as there is little chance for robots to sense their interaction partner’s internal characteristics, let alone influence these. Accordingly, robots deployed in public space will have to be able to live up to the expectations of many diverse individuals, without knowing who they might interact with next.

## 4 Conclusion

The proposed framework forms a generalisable, viable approach that allows for a wide range of applications, not limited to the investigation of embodied agents. Application areas furthermore include laboratory experiments as well as observational field studies, exploring the different methods for data acquisition. Guidance for defining and measuring compliance and reactance has been given, along with design implications addressing each step of the framework. In summary, our model proposes that in order to maximise the probability of compliance with a cue and to avoid reactance, it is necessary to aim for a high probability of perception, a high probability of comprehension and to prevent feelings of anger and negative cognitions. It outlines a novel approach to evaluating a new technology using the concepts of compliance and reactance as behaviours and affective constructs.

## Data Availability

The original contributions presented in the study are included in the article/Supplementary Material, further inquiries can be directed to the corresponding author.

## Abbreviations

HRI, human-robot interaction; perc, perception; compr, comprehension; acc, according to cue; cont, contrary to cue; compl, compliant; reac, reactant.
